# Seroprevalence and associated factors of Hepatitis B virus among diabetic adult patients attending at Haramaya General Hospital, Eastern Ethiopia

**DOI:** 10.3389/fpubh.2025.1454044

**Published:** 2025-05-02

**Authors:** Adnan Abdi, Firayad Ayele, Jemal Mohammed, Desalegn Admassu Ayana

**Affiliations:** ^1^Haramaya General Hospital, Haramaya, Ethiopia; ^2^School of Medical Laboratory Sciences, College of Health and Medical Science, Haramaya University, Harar, Ethiopia

**Keywords:** Hepatitis B virus infection, Seroprevalence, diabetic mellitus, Hepatitis B screening, Eastern Ethiopia

## Abstract

**Background:**

Hepatitis B virus (HBV) infection and diabetes mellitus are both significant public health concerns that substantially impact global morbidity and death. However, there is currently limited information available on the prevalence of HBV infection among diabetic patients in eastern Ethiopia. Therefore, this study aimed to determine the seroprevalence and associated factors of the Hepatitis B virus among diabetic adult patients at Haramaya General Hospital in Eastern Ethiopia from August 8 to August 30, 2021.

**Methods:**

An institution-based cross-sectional study was done among 365 diabetic patients. Study participants were chosen using a consecutive sampling technique. Data on sociodemographic characteristics and other associated factors were collected using a structured questionnaire. A blood sample was drawn from each participant, and the serum was separated and tested for HBsAg status using the Rapid Test Kit (ACON, USA). The data was entered into Epi Data version 4.6 software and analysed using SPSS version 25. Bivariate and multivariable logistic regression analyses were performed to examine the association between the outcome variable and predictor factors. A *p*-value less than 0.05 was considered statistically significant.

**Results:**

A total of 365 individuals with diabetes took part in this study. The overall seroprevalence of hepatitis B surface antigen among these patients was 7.4% (95% CI = 4.71–10.08). Among the total of 365, about 243 individuals (66.3%) were male, and the participants had a mean age of 42.24 ± 10.2 years. The only significant risk factor for Hepatitis B virus (HBV) coinfection among the diabetic patients was having multiple sexual partners [AOR = 2.92, 95% CI: 1.2–7.08].

**Conclusion:**

This study found an intermediate prevalence of Hepatitis B virus (HBV) infection among people with diabetes. Multiple sexual partners were strongly associated with an increased likelihood of HBV infection. Based on these findings, it is recommended to implement routine HBsAg screening for diabetic patients during regular medical visits to enable earlier detection and timely treatment. The government also should increase vaccination coverage for diabetic patients. Healthcare facilities, regional authorities, and experts should also teach diabetes patients about hepatitis B transmission routes and prevention strategies, with an emphasis on modifiable variables.

## 1 Introduction

Hepatitis B Virus (HBV) infection remains a prominent worldwide health concern, affecting populations worldwide (1). It is important to note that humans are the sole natural hosts for this virus. Despite the presence of an effective vaccine, HBV continues to present a substantial and ongoing global challenge, particularly in developing countries (2). Furthermore, diabetes is a common and complex metabolic disease marked by excessively elevated blood glucose levels. This chronic, non-communicable illness has a high mortality risk in both developed and developing countries (3). The prevalence of diabetes mellitus and HBV infection varies among diverse ethnic groups, with higher rates observed in Asian Americans and sub-Saharan Africans in contrast to Pacific Islanders (4).

In 2019, it was estimated that approximately 296 million people globally had a chronic hepatitis B infection. Additionally, there were approximately 1.5 million new infections reported each year. Tragically, hepatitis B caused approximately 820,000 deaths in 2019, with the main causes being complications like cirrhosis and liver cancer and Hepatitis B is recognized as the 10th important cause of death globally (5). These alarming figures establish hepatitis B as the 10th leading cause of death worldwide (6). On the other hand, diabetes presents a substantial mortality risk in both developed and developing nations, resulting in approximately 4.2 million deaths annually (7). These statistics underscore the significant global impact of both hepatitis B and diabetes on public health. In Ethiopia, the total prevalence of Hepatitis B Virus (HBV) infection ranges from 4.7 to 16.8%, depending on the presence of Hepatitis B surface antigen. Furthermore, the prevalence of at least one positive marker for HBV infection is expected to range between 70 and 76.38% (8–12).

Diabetes Mellitus has been linked to infection with the Hepatitis B Virus (HBV) (13) through two main pathways. Firstly, HBV can directly infect and destroy insulin-producing pancreatic *β*-cells, impairing insulin production. Secondly, HBV can trigger or contribute to an autoimmune response targeting *β*-cells, leading to their destruction and the development of type IDM (14). Understanding the link between hepatitis viruses and diabetes is crucial for developing precise preventive measures and treatments (15). The association between diabetes and HBV infection is still a subject of debate, as there are multiple factors involved. Further research is required to gain a better understanding of the potential relationship between the two (16).

Despite the significant health burdens posed by both Hepatitis B virus (HBV) and diabetes, there is a notable lack of comprehensive research specifically examining the prevalence of HBV among diabetic patients in Eastern Ethiopia. Existing studies have focused on HBV or diabetes in isolation, leaving a critical gap in understanding how these two health issues intersect within this population. Furthermore, the unique socio-economic and healthcare contexts of Eastern Ethiopia necessitate tailored research to inform targeted public health strategies. Addressing this gap is essential for developing effective interventions aimed at reducing HBV-related morbidity and mortality among diabetic patients in the region. Therefore, this study aimed to determine the magnitude of Hepatitis B virus infection and associated factors among diabetes patients.

## 2 Materials and methods

### 2.1 Study area, design, and period

A cross-sectional study was done from August 8 to August 30, 2021, at Haramaya General Hospital, situated in the eastern Hararghe Zone of Ethiopia. Haramaya town is located roughly 426 kilometers from Addis Abeba and has an estimated area of 467 square kilometers. The hospital was established in 2005 G.C. through the upgrade of a health center, and in 2010 G.C., it further developed into a General Hospital. As of 2012, the hospital had a workforce consisting of 367 healthcare workers and 56 administrative staff members. (source from human resource office at HGP, 2012).

### 2.2 Population

The source population for the study comprised diabetic patients who attended the diabetes clinic at Haramaya General Hospital. The study population included all individuals with a confirmed diagnosis of diabetes who visited the clinic during the study period.

### 2.3 Inclusion, and exclusion criteria

All adult type 2 diabetes patients who visited Haramaya General Hospital’s diabetic clinic during the study period were included. Individuals who were recognized as diabetic patients throughout the research period but refused to provide informed permission, individuals who were very unwell and unable to provide a blood sample, and diabetic patients under the age of 18 were also excluded.

### 2.4 Sample size and sampling technique

Taking into account the 8.5% reported prevalence of Hepatitis B virus among diabetes patients from previous research at the University of Gondar Referral Teaching Hospital in northwest Ethiopia (17), the sample size for this study was calculated using the single population proportion formula. The parameters considered were:

Confidence level: Zα/2 = 1.96

Prevalence: *p* = 0.085

Margin of error: d = 0.03

The formula used is: $n=\frac{{\left(Z\alpha /2\right)}^{2}p\;\left(1-p\right)}{d^{2}}$

$n=\frac{{\left(1.96\right)}^{2}0.085\left(1-0.085\right)}{0.03^{2}}$

*n* = 332.

After considering 10% non-response rate, the final sample size is adjusted to 365. Participants for the study were then selected using a convenience sampling technique until the full sample size was achieved.

### 2.5 Data collection instrument and data collectors

The data was collected using a standardized questionnaire. The questionnaire includes sociodemographic information such as age, sex, residence, occupation, educational status, and marital status. Behavioral factors such as multiple sexual partners and alcohol drinking were also assessed. The questionnaire also includes health-related factors questions such as history of sexually transmitted diseases/infections (STD/STI), blood transfusion, HBV vaccination, and family history of hepatitis. The data was then collected by two certified medical laboratory technicians, who were supervised by the lead investigator and coauthors.

### 2.6 Sample collection, handling, and transportation

A 3–5 mL sample of blood was drawn from each individual, and the samples were kept undisturbed to promote coagulation. The resulting serum was then transferred into a clean and dry cryovial and stored at a temperature of −20°C until further use. Screening for HBV surface antigen (HBsAg) was conducted using a rapid test kit (ACON Test cassette; USA). The assay process strictly followed standard procedures.

### 2.7 Laboratory method/ACON HBsAg rapid test

#### 2.7.1 Principle

The ACON HBsAg Rapid Test Cassette (Serum/Plasma) is designed for the qualitative detection of HBsAg in serum or plasma specimens using a combination of monoclonal and polyclonal antibodies. This lateral flow immunoassay generates a colored line in the test region (T) to indicate a positive result, while the control line (C) confirms that the test was conducted properly. The test cassette contains anti-HBsAg particles and antibodies coated on the membrane (18).

#### 2.7.2 Directions for use

To perform the test, bring the cassette and specimen to room temperature, add two drops of the specimen to the sample pad, and start the timer. Results should be read at 10 min, with no interpretations made after 30 min. A positive result is indicated by two distinct lines (C and T), while a negative result shows one line in the control region (C) only. If no control line appears, the test is invalid, and a new cassette should be used (18).

#### 2.7.3 Limitations of the test

The test is for *in vitro* diagnostic use only and should not be the sole criterion for diagnosing Hepatitis B, as it cannot detect HBsAg levels below 0.79 ng/mL. Negative results do not exclude the possibility of Hepatitis B infection (18).

#### 2.7.4 Performance characteristics

The ACON HBsAg Rapid Test demonstrates impressive performance characteristics, with a sensitivity of 100%, supported by a confidence interval ranging from 98.02 to 100%. Additionally, the test exhibits a specificity of 100%, with a confidence interval between 98.81 and 100%. Overall, the test shows an agreement of 99.6%, with a confidence interval spanning from 99.25 to 100%, indicating its reliability in detecting HBsAg in serum or plasma specimens (18).

### 2.8 Data quality assurance

The initial questionnaires were written in English. They were then translated into the local languages of Afan Oromo and Amharic by an impartial translator to ensure the study participants could fully understand the content. After the translation, the questionnaires were retranslated back into English to verify their consistency with the original English version. Data collectors and supervisors underwent comprehensive training on data collection procedures to ensure accurate and standardized data collection. To ensure the appropriateness and understandability of the questionnaires, a pretest was conducted on 15 diabetic patients attending Jugal Hospital. This allowed for any necessary corrections or adjustments to be made to the questionnaires before the actual data collection process. The collected data was checked by supervisors and principal investigators for consistency and completeness. Completed questionnaires were assigned unique codes for identification purposes. Qualified laboratory professionals were responsible for collecting blood specimens, and following standardized procedures for sample collection, storage, transportation, and the analytical process. In addition to ensure quality and accuracy, all test batches of rapid test strips or kits were verified using known negative and positive controls. Quality control is built into the test, as the control line (C) verifies adequate specimen volume and correct procedure. It is recommended to periodically test both positive and negative controls to ensure test performance. The whole procedure adhered closely to the pre-analytical, analytical, and post-analytical stages of quality assurance, as well as the manufacturer’s Standard Operating Procedures (SOPs). Internal quality control methods were adopted to prevent measurement bias during the testing of serum (plasma). The Hepatitis B quick test kits were appropriately stored at room temperature, specifically at 25°C.

### 2.9 Statistical analysis

The collected data was carefully reviewed to ensure the information is full and consistent. It was then coded and entered into Epi-data version 4.6. The data was then exported and analyzed using the Statistical Package for the Social Sciences (SPSS) version 25 software The data was summarized using descriptive statistics, and the findings were presented as texts, tables, and graphs. The data was described using summary measurements such as percentages, means, and medians. Bivariate logistic regression analysis was performed to evaluate the connections between variables, and *p* values of less than or equal to 0.25 were considered for multivariable logistic regression. The existence of relationships was established with adjusted odds ratios and 95% confidence intervals. A *p*-value of less than 0.05 was considered statistically significant.

### 2.10 Ethical consideration

The study protocol was evaluated and approved by the institutional ethics review committee (IHRERC/154/2021). The principal investigators received letters of support from Haramaya General Hospital. Participants were briefed about the study’s aims, methods, possible risk, and benefits. Participants were informed that they might decline participation or withdraw from the research at any moment without penalty. Participant confidentiality was ensured by eliminating names and identifiers. All participants provided informed, voluntary written consent. To protect themselves from COVID-19, participants were urged to wear face masks and use hand sanitizer during the trial. Participants who tested positive for hepatitis B were sent to the outpatient section to see a doctor for proper treatment.

## 3 Results

### 3.1 Socio-demographic characteristics of the study participants

**Figure 1:**
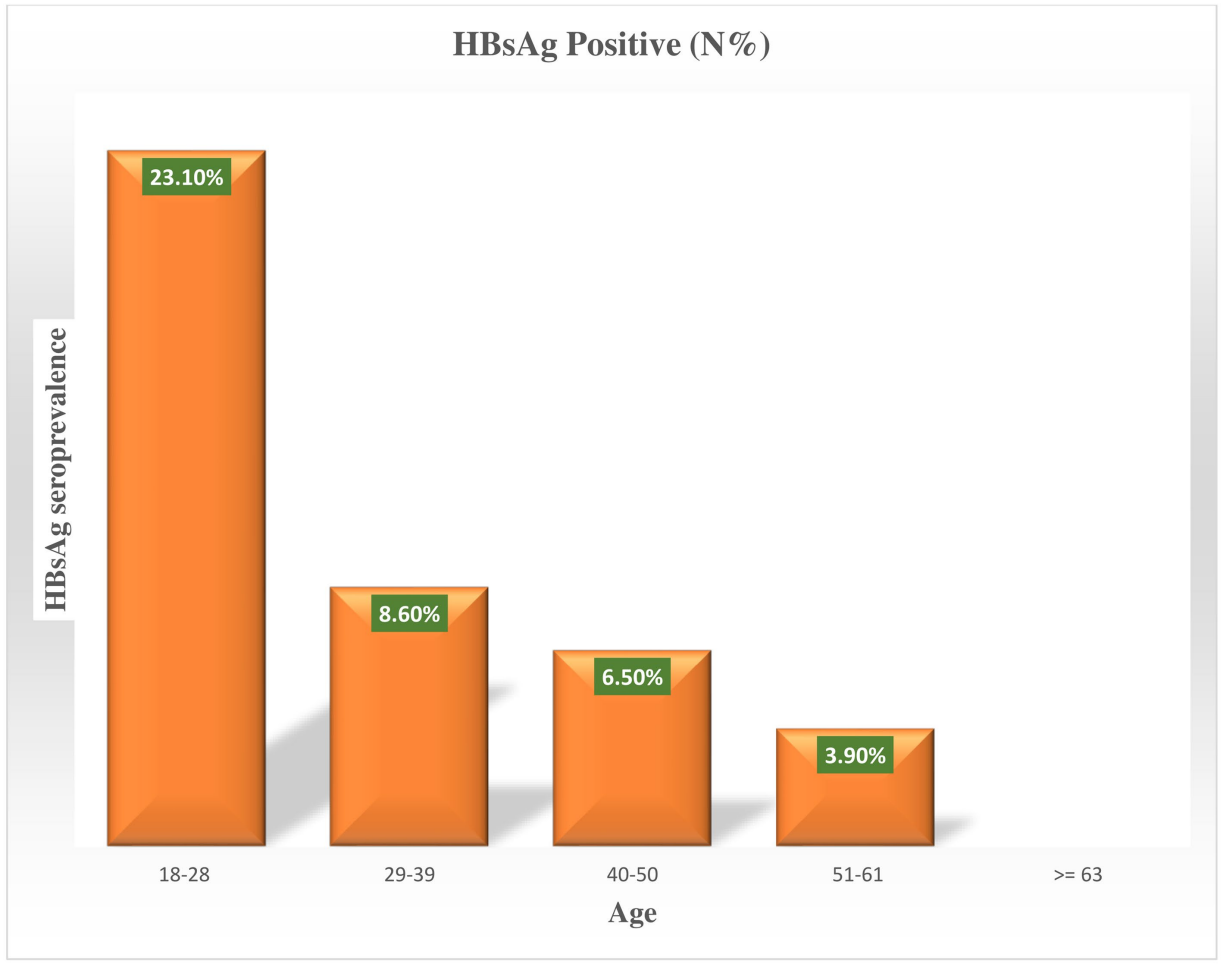
Prevalence of Hepatitis B virus by age among diabetic patients attending Haramaya General Hospital, Ethiopia, 2021.

In this study, a total of 365 diabetic patients participated, with a 100% response rate. The majority of diabetic patients, 47.9%, were found to be between the ages of 29–39 (Figure 1).

**Figure 2:**
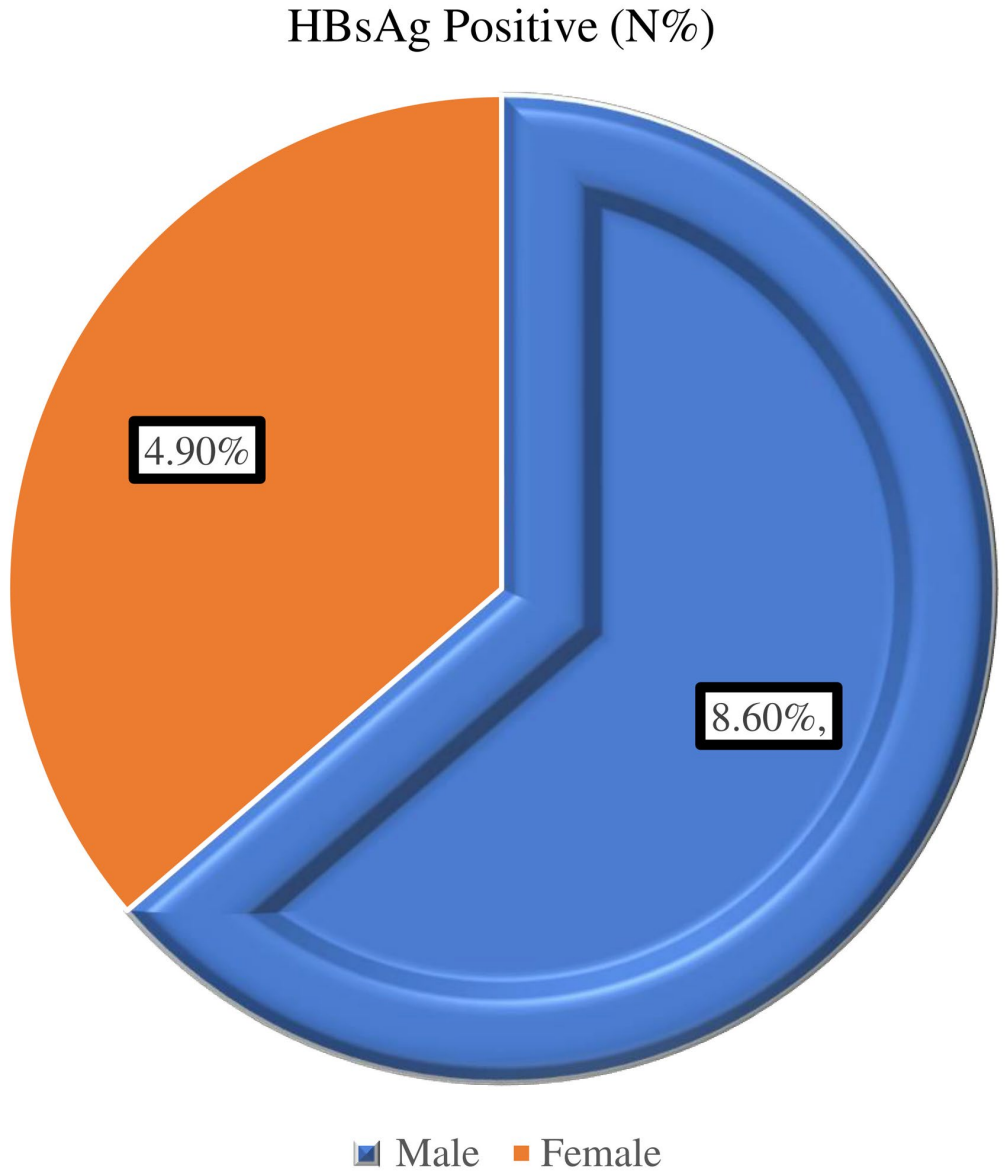
Prevalence of Hepatitis B virus by sex among diabetic patients attending Haramaya General Hospital, Ethiopia, 2021.

Concerning age, the majority of participants were men, comprising 243 individuals (66.5%). The findings also indicate a higher percentage of HBsAg positivity in males compared to females within the sample population (Figure 2).

In terms of marital status, most participants, 298 (81.6%), were married. Additionally, more than half of the participants, 241 (66%), had no formal education (Unable to Read and write). The majority of the diabetic patients, 291 (79.7%), were employed in the private sector. Concerning residence, 239 (65.4%) of the participants were from urban areas (Table 1).

**Table 1:** Socio-demographic characteristics of diabetes patients attending Haramaya General Hospital, Ethiopia, 2021(n = 365).

Variables		HBsAg seroprevalence	
		Positive (*N*%)	Negative (*N*%)
Marital status	Married	21 (7.4%)	276 (92.6%)
	Single	5 (8.2%)	45 (91.8%)
	Divorced	–	8 (100.0%)
	Widowed	1 (10.0%)	9 (90.0%)
Educational status	Unable to read and write	16 (6.6%)	225 (93.4%)
	Read and write	5 (8.9%)	51 (91.1%)
	Primary school	4 (10.8%)	33 (89.2%)
	College	2 (6.5%)	29 (93.5%)
Occupation	Private employee	23 (7.9)	268 (92.1%)
	Government employee	4 (6.5%)	58 (93.5%)
	Driver	–	5 (100%)
	Merchant	–	7 (100%)
Residence	urban	16 (6.7%)	223 (93.3%)
	Rural	11 (8.7%)	115 (91.3%)

### 3.2 Clinical and behavioral characteristics of the study participants

In this study, the majority of the study participants, 129 (35.3%), were active smokers, while 9 (2.46%) reported alcohol users. Out of the total 365 participants, 69 (18.9%) had a history of tattooing, 58 (15%) reported having multiple sexual partners, 110 (30.1%) had a history of sexually transmitted infections (STIs) or sexually transmitted diseases (STDs), and 42 (11.50%) had a history of blood transfusion. Approximately 73 (20%) of the study participants had a family history of Hepatitis (Table 2).

**Table 2:** Clinical and behavioral characteristics of diabetes patients attending Haramaya General Hospital, Ethiopia, 2021 (*n* = 365).

Variables		HBsAg seroprevalence	
		Positive *N* (%)	Negative *N* (%)
Alcohol consumption	Yes	1 (11.1%)	8 (88.9%)
	No	26 (7.3%)	330 (92.7%)
Active smoker	Yes	9 (7%)	120 (93%)
	No	18 (7.6%)	218 (92.4%)
History of tattooing	Yes	8 (11.6%)	61 (88.4%)
	No	19 (6.4%)	277 (93.6%)
History of multiple sex partner	Yes	11 (19%)	47 (81%)
	No	16 (5.2%)	291 (94.8%)
History of STI/STD	Yes	14 (12.7%)	96 (87.3%)
	No	13 (5.1%)	242 (94.9%)
History of blood transfusion	Yes	5 (11.9)	37 (88.1%)
	No	22 (6.8%)	301 (93.2%)
History of hypertension	Yes	16 (12.6%)	111 (87.4%)
	No	11 (4.6%)	227 (95.4%)
HBV vaccinated	Yes	3 (9.7%)	28 (90.3%)
	No	24 (7.2%)	310 (92.8%)
Family history of hepatitis	Yes	9 (12.3%)	64 (87.7%)
	No	18 (6.2%)	274 (93.8%)

STI/STD = transmitted infections/sexually transmitted diseases.

### 3.3 Seroprevalence of Hepatitis B virus infection

**Figure 3:**
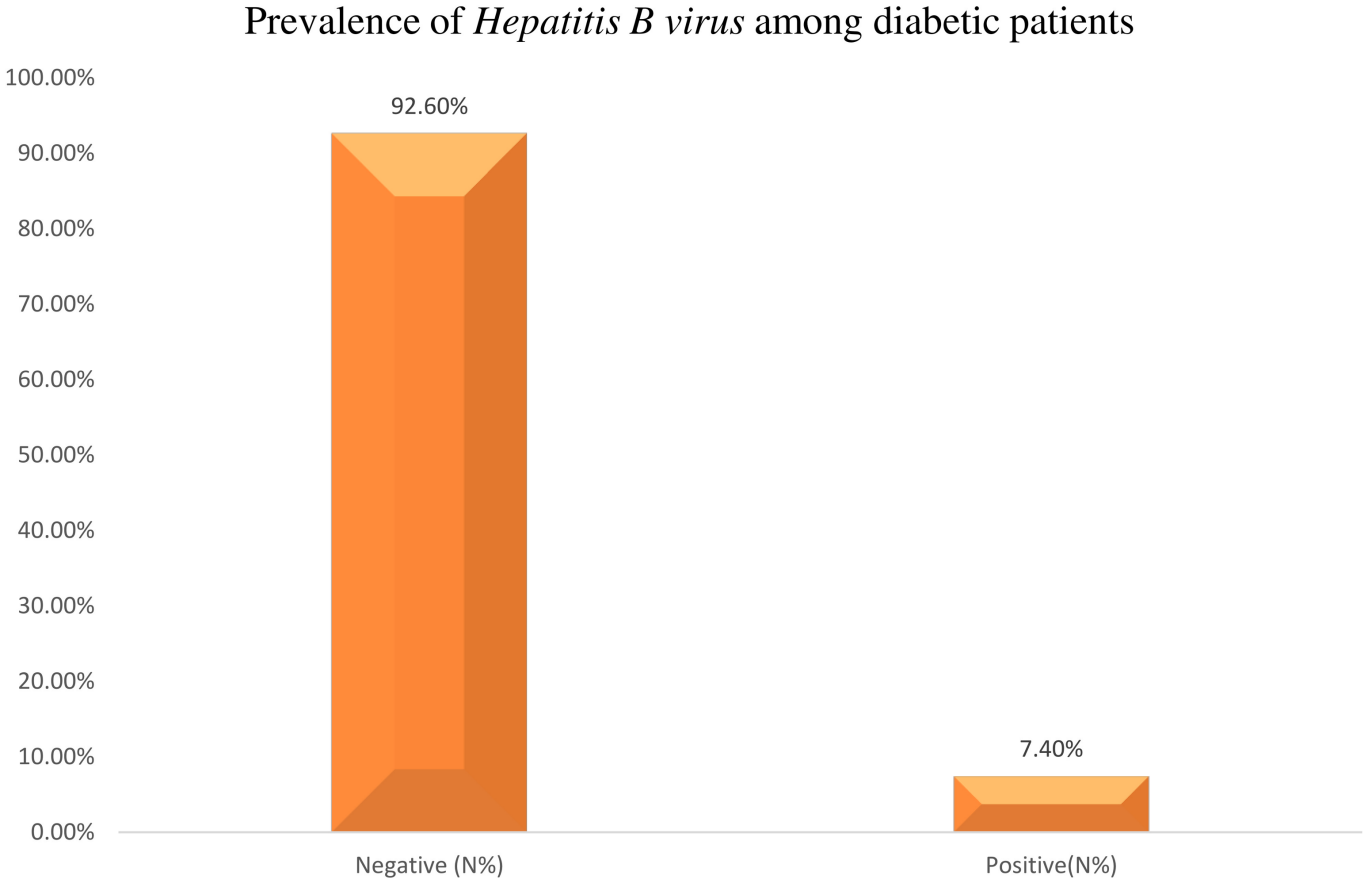
Prevalence of Hepatitis B virus among diabetic patients of attending Haramaya General Hospital, Ethiopia, 2021.

In this particular study, 365 diabetic patients were included and screened for HBsAg. Out of this, 27 diabetic patients tested positive for HBsAg. Consequently, the overall seroprevalence of HBV infection among diabetic patients was calculated to be 7.4% (95% CI = 4.71–10.08) (Figure 3).

### 3.4 Factors associated with Hepatitis B virus among diabetic patients

In the bivariate analysis, sex, having a history of multiple sexual partners, history of STI/STD, history of blood transfusion, hypertension, and having a family history of Hepatitis were factors associated with Hepatitis B virus among diabetic patients with a *p* < 0.25. All variables with a *p*-value less than or equal to 0.25 in the bivariate regression analysis were candidates for the multivariable model. Following the adjustment for confounding variables in the final model of multivariable logistic regression, the analysis revealed that diabetic patients who had a history of multiple sexual partners had 2.92 times higher odds of being infected with the Hepatitis B virus [AOR = 2.92, 95% CI: 1.2–7.08] (Table 3).

**Table 3:** Factors associated with Hepatitis B virus among diabetic patients Haramaya hospital, Ethiopia, 2021.

Variables		HBsAg seroprevalence		COR (95% CI)	AOR (95% CI)	*p*-value
		Positive *N* (%)	Negative *N* (%)			
Sex	Male	21 (8.6%)	222 (91.4%)	1.82 (0.71–4.65)	1.78 (0.67–4.67)	0.24
	Female	6 (4.9%)	116 (95.1%)	1.00	1.00	
History of multiple sex partners	Yes	11 (19%)	47 (81%)	4.25 (1.86–9.73)	2.92 (1.2–7.08)	0.018*
	No	16 (5.2%)	291 (94.8%)	1.00	1.00	
History of STI/STD	Yes	14 (12.7%)	96 (87.3%)	2.71 (1.23–5.98)	1.83 (0.78–4.25)	0.16
	No	13 (5.1%)	242 (94.9%)	1.00	1.00	
History of blood transfusion	Yes	5 (11.9)	37 (88.1%)	1.84 (0.66–5.17)	1.48 (0.49–4.50)	0.48
	No	22 (6.8%)	301 (93.2%)	1.00	1.00	
History of hypertension	Yes	16 (12.6%)	111 (87.4%)	2.97 (1.33–6.66)	2.18(0.92–5.18)	0.075
	No	11 (4.6%)	227 (95.4%)	1.00	1.00	
Family history of hepatitis	Yes	9 (12.3%)	64 (87.7%)	2.14 (0.91–4.98)	1.2 (0.48–3)	0.68
	No	18 (6.2%)	274 (93.8%)	1.00	1.00	

STI/STD = transmitted infections/sexually transmitted diseases ∗ = variable is statistically associated with the dependent variable in Multivariate, AOR = adjusted odds ratio, CI = Confidence Interval.

## 4 Discussion

The global prevalence of Hepatitis B virus (HBV) infection can be categorized into 3 main groups based on the level of endemicity: high-endemicity regions with a prevalence greater than 8%, intermediate-endemicity regions with a prevalence ranging from 2 to 7%, and low-endemicity regions with a prevalence below 2%. However, it’s important to note that these classifications provide a general understanding, and actual prevalence rates may vary within countries and regions due to various factors. In this case, the study area is close to high endemicity areas according to WHO guidelines (19).

In current study, the total prevalence of Hepatitis B virus among diabetic patients was 7.4% (95% CI = 4.71–10.08), which is in line with findings from similar studies conducted in Gondar, Northwest Ethiopia (8.5%) (17), China (5.32%) (20), Iran (7.6%) (21), and São Paulo, Brazil (8.2%) (7) but lower than a study in the USA that reported a prevalence of (11%) (22), Nigeria 13.3% (23); the discrepancy could be due to variations in sample size and sampling techniques across the different studies. In addition, it could be laboratory test method, sampling techniques and epidemiological distribution of HBV infection. But it is found to be higher than studies done in the North-eastern Democratic Republic of Congo (3.4%) (24), Woldiya General Hospital, Ethiopia (3.7%) (12), Brazil (0.55) (25), Iraqi Kurdistan (2.13%) (26), Nigeria (3.85%) (20), South Korea (3.8%) (27) and of Iraqi Kurdistan (2.13%) (26). This discrepancy might be due to traditional practice, immunity, sample size variations, and other socioeconomic characteristics. Additionally, the methods of detection used may also contribute to this difference. However, the most significant factor is likely the impact of vaccination. The introduction and widespread adoption of effective vaccines against Hepatitis B virus would be expected to reduce its overall prevalence in the population, leading to the observed differences in reported rates between the study groups (20, 28). The CDC and ACIP also recommend hepatitis B vaccination for unvaccinated adults under 60 with diabetes to protect this high-risk population (29).

In this study, diabetic patients who had multiple sexual partners had 2.92 times the odds of being infected with Hepatitis B virus compared to those who practiced monogamy or had a single sexual partner. This finding is reasonably acceptable because the Hepatitis B virus is sexually transmitted from one individual to another (30–32), which aligns with the study findings reported from Gondar, Northwest Ethiopia (17). Additionally, unvaccinated diabetic patients who have multiple sexual partners are at increased risk of transmitting the virus (33). However, the study conducted in Woldiya, Ethiopia showed no significant association between having multiple sexual partners and Hepatitis B virus infection. This discrepancy might be due to the reason that some people used condoms during sexual intercourse which can reduce the risk of Hepatitis B transmission (34).

In this study, key variables like smoking and transfusion history were not linked to the outcome variable. However, these factors are known to be strongly associated with HBV infection. Research indicates that among individuals with HBV, smoking more than 20 cigarettes per day is a significant risk factor that worsens the progression to liver cirrhosis (35). Donating blood saves lives, but blood transfusions also carry the risk of transmitting transfusion-transmissible infections, particularly HIV, HBV, HCV, and syphilis. HBV is a significant concern, as it can be transmitted if blood donors test positive for HBVsAg. However, the risk of transmission can be reduced through effective screening and donor selection processes. Therefore, the screening practices and selection methods may explain why this variable is not associated with the outcome variable (36, 37).

### 4.1 Strengths and limitations of the study

The study highlighted various strengths and drawbacks that should be considered. One of the primary strengths is its focus on a crucial public health issue: the seroprevalence of Hepatitis B virus (HBV) among diabetic patients. This population is at an increased risk for various infections, making the findings particularly significant for public health interventions and resource allocation.

However, the study also faced notable limitations. While the limitations are acknowledged, they lack sufficient depth and specificity. Due to resource constraints, the study was unable to perform comprehensive serological testing, which included the evaluation of key markers such as anti-HBs, anti-HBc, and IgM anti-HBc. These markers are essential for accurately diagnosing HBV infection and identifying the stage of infection. The absence of these advanced serological markers significantly impacts the study’s findings, as it limits the ability to provide a complete immunological profile of participants. Additionally, the study did not assess viral load in HBsAg-positive diabetic patients, which is critical for understanding the dynamics of HBV infection and its potential impact on patient management and treatment outcomes.

Furthermore, the study’s reliance on self-reported data introduces challenges associated with recall bias, particularly concerning sensitive variables such as sexual behavior. Participants may struggle to accurately recall past behaviors or may alter their responses due to social desirability bias, leading to underreporting or overreporting of risk-related behaviors. This limitation could skew the data and affect the reliability of the conclusions drawn.

Overall, while the study highlights an important area of research, the limitations emphasize the need for more comprehensive approaches in future studies to better understand HBV in this vulnerable group. Addressing these limitations with greater specificity will enhance the rigor of the research and provide clearer insights into HBV seroprevalence among diabetic patients.

## 5 Conclusion and recommendation

This study found an intermediate prevalence of Hepatitis B virus (HBV) infection among diabetic patients, with multiple sexual partners identified as a significant associated factor. Based on these findings, several specific and actionable recommendations are proposed. First, it is crucial to implement routine HBsAg screening for diabetic patients at least once a year, with high-risk individuals, such as those with multiple sexual partners or a history of injection drug use, screened every 6 months. Additionally, integrating HBV vaccination into standard diabetes care protocols is essential; healthcare providers should ensure that all newly diagnosed diabetic patients receive information about the HBV vaccine and are vaccinated if they are not already immune.

To address cultural barriers to HBV prevention in Ethiopian communities, healthcare providers should undergo cultural competency training to better understand and communicate effectively with patients. Community awareness campaigns highlighting the importance of HBV prevention, vaccination, and regular screening should be launched, utilizing local languages and culturally relevant materials to increase outreach. Collaborating with community leaders and influencers can further promote HBV prevention and vaccination, helping to overcome stigma and encouraging community members to seek necessary care.

Enhancing access to healthcare services, especially through mobile clinics and outreach programs in rural or underserved areas, will ensure that diabetic patients can access HBV screening and vaccination easily. Establishing support groups for diabetic patients can also foster peer support and provide a safe space to discuss HBV prevention and management. Furthermore, a robust follow-up system for diabetic patients who test positive for HBV should be developed, including regular monitoring, access to antiviral therapies, and referrals to specialists as needed. Finally, conducting further research to identify specific cultural barriers to HBV prevention will inform more effective intervention strategies. By implementing these targeted recommendations, we aim to enhance the prevention and management of HBV among diabetic patients, reduce related complications, and improve health outcomes within the broader population.

## Data Availability

The datasets presented in this study can be found in online repositories. The names of the repository/repositories and accession number(s) can be found in the article/supplementary material.
